# Clinical Validation of Two Recombinase-Based Isothermal Amplification Assays (RPA/RAA) for the Rapid Detection of African Swine Fever Virus

**DOI:** 10.3389/fmicb.2020.01696

**Published:** 2020-07-21

**Authors:** Xiaoxu Fan, Lin Li, Yonggang Zhao, Yutian Liu, Chunju Liu, Qinghua Wang, Yaqin Dong, Shujuan Wang, Tianying Chi, Fangfang Song, Chengyou Sun, Yingli Wang, Dengchuriya Ha, Yang Zhao, Jingyue Bao, Xiaodong Wu, Zhiliang Wang

**Affiliations:** ^1^National Reference Laboratory for African Swine Fever, National Surveillance and Research Center for Exotic Animal Diseases, National Surveillance and Research Center for Exotic Animal Diseases, China Animal Health and Epidemiology Center, Qingdao, China; ^2^Livestock Disease Surveillance Laboratory, China Animal Health and Epidemiology Center, Qingdao, China; ^3^Vocational and Technical College, Inner Mongolia Agricultural University, Hohhot, China; ^4^College of Veterinary Medicine, Inner Mongolia Agricultural University, Hohhot, China

**Keywords:** African swine fever virus, recombinase polymerase amplification, recombinase aided amplification, rapid isothermal amplification, clinical validation

## Abstract

African swine fever (ASF), caused by African swine fever virus (ASFV), is a devastating infectious disease of domestic pigs and wild boars, and has tremendous negative socioeconomic impact on the swine industry and food security worldwide. It is characterized as a notifiable disease by World Organisation for Animal Health (OIE). No effective vaccine or treatment against ASF has so far been available. Early detection and rapid diagnosis are of potential significance to control the spread of ASF. Recombinase-based isothermal amplification assay, recombinase polymerase amplification (RPA) developed by TwistDx (Cambridge, United Kingdom) or recombinase-aided amplification (RAA) by Qitian (Wuxi, China), is becoming a molecular tool for the rapid, specific, and cost-effective identification of multiple pathogens. In this study, we aim to investigate if RPA/RAA can be a potential candidate for on-site, rapid and primary detection of ASFV. A panel of 152 clinical samples previously well-characterized by OIE-recommended qPCR was enrolled in this study, including 20 weak positive (Ct value ≥ 30) samples. This panel was consisted of different types, such as EDTA-blood, spleen, lung, lymph node, kidney, tonsil, liver, brain. We evaluated two recombinase-based isothermal amplification assays, RPA or RAA, by targeting the ASFV *B646L* gene (p72), and validated the clinical performance in comparison with OIE real-time PCR. Our result showed that the analytical sensitivity of RPA and RAA was as 93.4 and 53.6 copies per reaction, respectively at 95% probability in 16 min, at 39°C. They were universally specific for all 24 genotypes of ASFV and no cross reaction to other pathogens including Classical swine fever virus (CSV), Foot-and-mouth disease virus (FMDV), Pseudorabies virus, Porcine circovirus 2 (PCV2), Porcine Reproductive and respiratory syndrome virus (PPRSV). The results on detection of various kinds of clinical samples indicated an excellent diagnostic agreement between RPA, RAA and OIE real-time PCR method, with the kappa value of 0.960 and 0.973, respectively. Compared to real-time PCR, the specificity of both RPA and RAA was 100% (94.40% ∼ 100%, 95% CI), while the sensitivity was 96.59% (90.36% ∼ 99.29%, 95% CI) and 97.73% (92.03% ∼ 99.72%, 95% CI), respectively. Our data demonstrate that the developed recombinase-based amplification assay (RPA/RAA), promisingly equipped with field-deployable instruments, offers a sensitive and specific platform for the rapid and reliable detection of ASFV, especially in the resource-limited settings for the purpose of screening and surveillance of ASF.

## Highlights

- Recombinase-based isothermal amplification assays (RPA/RAA) were specific and sensitive, with the detection limits of 93.4 and 53.6 copies per reaction at 95% probability in 16 min.
- Both RPA and RAA present good agreement with OIE real-time PCR for the detection of African swine fever virus (ASFV) with clinical samples.
- These methods, coupled with field-deployable platform, provide an easy, reliable, sensitive and specific tool for the rapid diagnosis of ASFV, particularly screening and surveillance for the early detection.

## Introduction

African swine fever (ASF), as a notifiable disease to the World Organization for Animal Health (OIE), is a highly contagious, viral pig disease caused by African swine fever virus (ASFV). ASFV has been currently classified to the genus *Asfivirus* of the family *Asfarviridae*. The genome of this large, complex double-stranded DNA virus (170–193 kbp) consists of 151–167 genes (Galindo and Alonso, 2017). So far, 24 different genotypes and 8 serogroups have been identified based on ASFV *B646L* gene (encoding the capsid protein p72) and *EP402R* gene (encoding the serotype-specific protein CD2v), respectively (Bastos et al., 2003; Malogolovkin et al., 2015a, b). ASF infection of domestic pigs and wild boars causes high fever, lethargy, digestive dysfunction, respiratory discharges, nasal discharges and abortion, with the mortality close to 100%. The diverse transmission modes include a sylvatic cycle between swine and arthropod vector (*Ornithodoros moubata* ticks), direct or indirect contact between susceptible animal and infected pigs, contaminated secretions (blood, feces, urine, mucus) or fomites (vehicles, equipment) (Dixon et al., 2019). To date, no effective vaccine or antiviral treatment has been developed, while the quarantine, depopulation and sanitation strategies remain the routine ways to control the spread of ASF.

African swine fever belongs to a transboundary animal disease. Since it was first described in Kenya in 1921 (Montgomery, 1921), ASFV has been found in other 25 African countries (Randriamparany et al., 2016). In the 1950s, ASF incursions were subsequently reported in European countries, including Portugal, Spain, France, Italy, Belgium, the Netherlands, Malta and the virus further spread to the Caribbean and South America (Brazil, Cuba, Haiti, Dominican Republic) (Ekue et al., 1989). Except for Sardinia, Madagascar and sub-Saharan African countries, all of the outbreaks were eradicated in the mid 1990s. In 2007, ASF was first reported in Georgia and continued its spread to the Trans-Caucasus region, parts of the Russian Federation and Eastern Europe, which has already affected Russia, Ukraine, Belarus, Azerbaijan, Armenia, Poland, Latvia, Lithuania, Estonia, Moldova, Czech Republic, Hungary, Bulgaria, Romania and Belgium through large geographic jumps (Sanchez-Cordon et al., 2018). In August 2018, ASF outbreak in China was first reported (Ge et al., 2018; Zhou et al., 2018), and the infections later occurred in other Asian countries, including Mongolia, Viet Nam, Cambodia, Democratic People’s Republic of Korea, Lao People’s Democratic Republic, Myanmar, The Philippines, Republic of Korea, Timor-Leste, Indonesia, Papua New Guinea, India^1^. The disease poses a serious threat and high hazard to the swine industry and food security worldwide.

**FIGURE 1:**
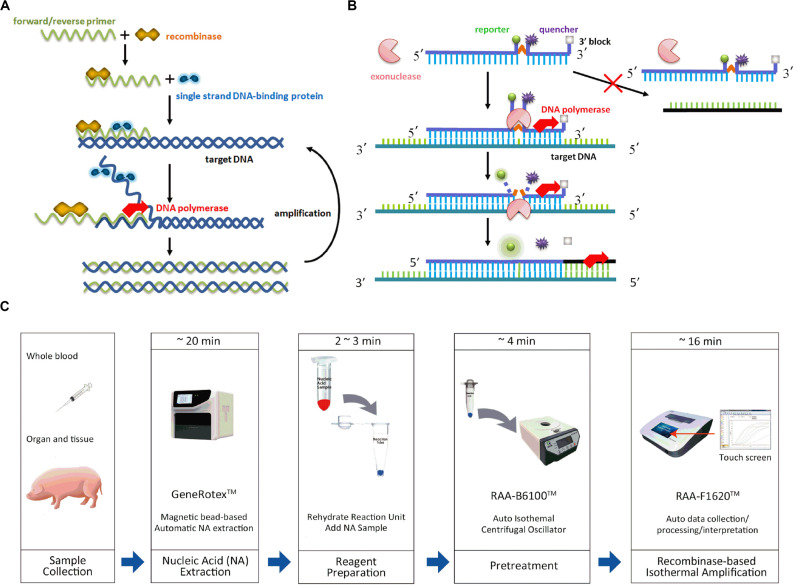
Schematic of recombinased-based assay and process of sampling, nucleic acid extraction and amplification. **(A)** Recombinased-based reaction mechanism. **(B)** Probe principle. **(C)** Whole process of recombinased-based (RPA/RAA) detection.

Polymerase chain reaction (PCR)-based diagnostic methods have been commonly applied for the detection of ASFV with high sensitivity and specificity, including conventional PCR and real-time PCR. Basically, its complex procedures consist of three steps, denaturation, annealing and extension, in multiple cycles, which require highly specialized equipment. However, they are costly, time-consuming and inappropriate for wide application in resource-limited laboratories or even in the field. Isothermal amplification has recently been introduced for the detection of various viruses (Congdon et al., 2019; Hu et al., 2019; Silva et al., 2019). The detection of ASFV with loop-mediated isothermal amplification (LAMP), for instance, shows concordance with real-time PCR but requires four or more primers, and primer design is typically complex (James et al., 2010; Wozniakowski et al., 2018). The development of alternative, recombinase-based isothermal amplification, i.e., recombinase polymerase amplification (RPA) developed by TwistDx (Cambridge, United Kingdom) (Li Y. et al., 2018), or recombinase-aided amplification (RAA) by Qitian (Wuxi, China) (Shen et al., 2019), advance nucleic acid-based tests as rapid, specific, diversified readout, and avoid the use of comprehensive thermal cyclers. It employs the recombinase and its cofactor to bind with oligonucleotide primers in search for homologous DNA. However, as the critical component in RPA, uvsX is the recombinase of T4 phage while the recombinant enzyme of RAA is obtained from *E. coli*, which can closely bind to primer DNA at room temperature. The strands then exchange after the recognition and single strand binding (SSB) protein combines with the parental strand to continue the amplification with template strand. DNA polymerase launches the template synthesis from 3′ –terminal of primers for the form of new duplex DNA. In this way, specific fragment is exponentially amplified as the cycle is repeated (Rohrman and Richards-Kortum, 2012) (Figure 1A).

Recombinase-based isothermal amplification is conducted at 37–42°C. The detectable amplification signal requires 10–20 min and can be detected by gel electrophoresis, real-time monitoring, or visualizing with lateral flow dipstick (LFD). The real-time detection incorporates a fluorogenic probe besides forward and reverse primers, and the reaction initiates based on the cleavage of probe at an abasic site [i.e., tetrahydrofuran (THF) or a dSpacer (a derivative of the THF) or a dR group (the deoxyribose of the abasic site via a C-O-C linker)] between the fluorophor and the quencher with *E. coli* exonuclease III or glycosylase/lyase (Li J. et al., 2018) (Figure 1B). The real-time detection has been applied for the rapid diagnosis of several viruses (HIV, hepatitis B virus, coxsackievirus, respiratory syncytial virus, porcine circovirus 2, small ruminant morbillivirus) (Crannell et al., 2014; Wang et al., 2017b; Chen et al., 2018; Li Y. et al., 2018; Shen et al., 2019), bacteria (Salmonella, Clostridium difficile, Escherichia coli, Vibrio parahaemolyticus, Campylobacter jejuni, Actinobacillus pleuropneumoniae) (Tsaloglou et al., 2015; Choi et al., 2016; Zhang et al., 2017; Geng et al., 2019; Li et al., 2019), parasites (Fasciola hepatica) (Cabada et al., 2017). However, there is a potential risk of aerosol contamination of amplicon during the visual read-out procedure by gel electrophoresis and lateral flow strip (Chou et al., 2011; Boot et al., 2013). Real-time assay in a relatively close system can perform successfully in both laboratories and open-air environment, which rules out the possible influence of dust from outside and contamination leading to false positive result. Our preliminary data showed a rapid, sensitive, specific diagnostic assay for ASFV by using real-time fluorescent RPA (Ha et al., 2017). In this study, we aim to validate the clinical performance of two recombinase-based isothermal amplification methods, RPA, RAA, and further compared those with OIE real-time PCR on the detection of field samples.

## Materials and Methods

### Recombinant Plasmids and Viruses

The partial sequence of major capsid protein p72 gene characterized for genotyping from 24 ASFV genotypes (GenBank accession number: AF302816, AM999764, AF270706, FJ528594, DQ250120, AF302818, AY494553, AF270711, AF302818, AF270705, AY351564, AF449463, AY351522, AY351543, AY351542, AY351555, AY494552, AY494551, DQ250119, DQ250122, DQ250127, DQ250109, DQ250125, DQ250117, KT795360, KY353989) were synthesized and inserted into pUC57 plasmid vector (Sangon, Shanghai, China). The concentration of DNA (AM999764) was determined as 8.3 × 10^10^ copies/μL by using Nanodropone (Thermo Fisher Scientific, Waltham, MA, United States). Classical swine fever virus (CSV) (Shimen), Foot-and-mouth virus (FMDV) (MYA98), Pseudorabies virus (Bartha K61), Porcine circovirus 2 (PCV2) (ZJ/C), Porcine reproductive and respiratory syndrome virus (PPRSV) (SDWH) infected samples or porcine genomic DNA were preserved in National Surveillance and Research Center for Exotic Animal Disease.

### Clinical Samples

Clinical samples, including EDTA-blood, spleen, lung, lymph node, kidney, tonsil, liver, brain, were collected from domestic pigs in China. These samples were firstly detected by real-time PCR as OIE recommended (King et al., 2003) at the National Reference Laboratory for ASF, China Animal Health and Epidemiology Center from September to December, 2018. Based on the result, in this study, a panel of 64 negative and 88 positive samples (including 20 weak positive Ct value ≥ 30) was adopted.

### Nucleic Acid Extraction

We employed the TaqMan PCR assay with internal amplification control for the detection of African swine fever virus, along with additional extraction controls before this stage, according to the previous study (King et al., 2003). First of all, we need to ensure that no problem happened during the extraction process and then OIE-qPCR was conducted. In brief, the total viral DNA was extracted from 200 μL of samples [EDTA-blood, supernatant tissue homogenates diluted 1:10 in phosphate-buffered saline (PBS, pH 7.4)] by using Magnetic beads pre-filled viral nucleic acid extraction kit (Tianlong Science and Technology, Xi’an, Shanxi, China) according to the manufacturer’s instructions (Li Y. et al., 2018). The total nucleic acid was eluted using dH_2_O water in a final volume of 100 μL and stored at −80°C until further use for all assays in this study.

### Real-Time PCR Assay

The real-time PCR assay was performed on Light Cycler 480 (Roche, Mannheim, Germany) according to the previous study as OIE recommended (King et al., 2003). The reactions were prepared as a 20 μL reaction volume containing 10 μL 2× super mix containing enzyme (Takara, Kusatsu, Japan), 0.8 μL forward primers, 0.8 μL reverse primers, 0.4 μL probe and 2 μL extracted DNA. The following thermal program was: reverse transcription at 95°C for 2 min and 40 cycles of amplification (15 s at 94°C and 1 min at 60°C).

### ASFV-Specific Primers and Probe for RPA/RAA

The primers and probe applied in RPA/RAA reactions was based on previous report (Ha et al., 2017). Forward primer, 5′-TTCCGTAACTGCTCATGGTATCAATCTTATCG-3′; Reverse primer, 5′-GATACCACAAGATCAGCCGTAGTGATAGAC-3′; Probe, 5′-GATACGTTAATATGACCACTGGGTTGGTAT-FAM-C-THF-T-BHQ1-CCGTGGCTTCAAAGC. The primers and probe were synthesized from Sangon (Shanghai, China).

### Real-Time RPA/RAA Conditions

Real-time RPA assay was performed in a 50 μL volume using the TwistAmp exo kit (TwistDx, Cambridge, United Kingdom). The reaction mixture included 29.5 μL rehydration buffer, 2 μL extracted DNA template, 2.1 μL forward primer (10 μM), 2.1 μL reverse primer (10 μM), 0.6 μL probe (10 μM), 11.2 μL dH_2_O, and 2.5 μL magnesium acetate (280 mM). Real-time RAA assay was performed in a 50 μL volume using the RAA nucleic acid amplification kit (fluorescence method, F00001) (Qitian, Wuxi, China). The reaction mixture included 25 μL rehydration buffer, 2 μL extracted DNA template, 2.1 μL forward primer (10 μM), 2.1 μL reverse primer (10 μM), 0.6 μL probe (10 μM), 15.7 μL dH_2_O and 2.5 μL magnesium acetate (280 mM). The RPA or RAA reaction mixture was firstly treated in isothermal vibration mixer (RAA-B6100, Qitian, Wuxi, China) at 39°C for 4 min (brief mix, centrifugation, vibration) and then incubated for 16 min at 39°C with real-time fluorometer (RAA-F1620, Qitian, Wuxi, China) to detect fluorescence (FAM) signal every 20 s. The criteria of threshold limit for positive results were determined as described previously (Li Y. et al., 2018).

### Analytical Sensitivity and Specificity of Real-Time RPA/RAA

To determine the detection limit of the real-time RPA/RAA, serial dilutions of recombinant plasmids with concentration including 8.3, 41.5, 83, 415, 830, 4150, 8300 copies/μL were prepared. Each concentration was assayed in eight replicates by the real-time RPA/RAA. The analytical specificity of ASFV real-time RPA/RAA was evaluated among other swine pathogens with similar clinical signs (CSV, FMDV, PRV, PCV2, PPRSV and DNA fragments from 24 ASFV genotypes (10^3^ ∼ 10^4^ copies/μL).

### Comparison of the Real-Time RPA/RAA Assay With Real-Time PCR Assay Using Clinical Samples

To explore the clinical performance of the real-time RPA/RAA assays in the detection of clinical specimen, 152 samples by veterinary service were collected during outbreaks of ASFV from September to December 2018. The performance of real-time RPA/RAA assays was compared to that of real-time PCR assay. The degree of agreement between the real-time RPA/RAA and real-time PCR assay results were measured with kappa value by using MedCalc software (MedCalc Software bvba, Ostend, Belgium).

### Statistical Analysis

Data in this study were presented as mean ± standard deviation. For the determination of the ASFV real-time RPA/RAA assay analytical sensitivity, a semi-log regression analysis (PRISM, Graphpad Software Inc., San Diego, CA, United States). The probit regression analysis using MedCalc Software (MedCalc Software bvba, Ostend, Belgium) was performed with data of eight replicates from serial dilutions to calculate the detection limit of the real-time RPA/RAA assay at a 95% probability level. At least triplicates were used in the experiment.

## Results

### Sensitivity and Specificity of ASFV Real-Time RPA/RAA

**FIGURE 2:**
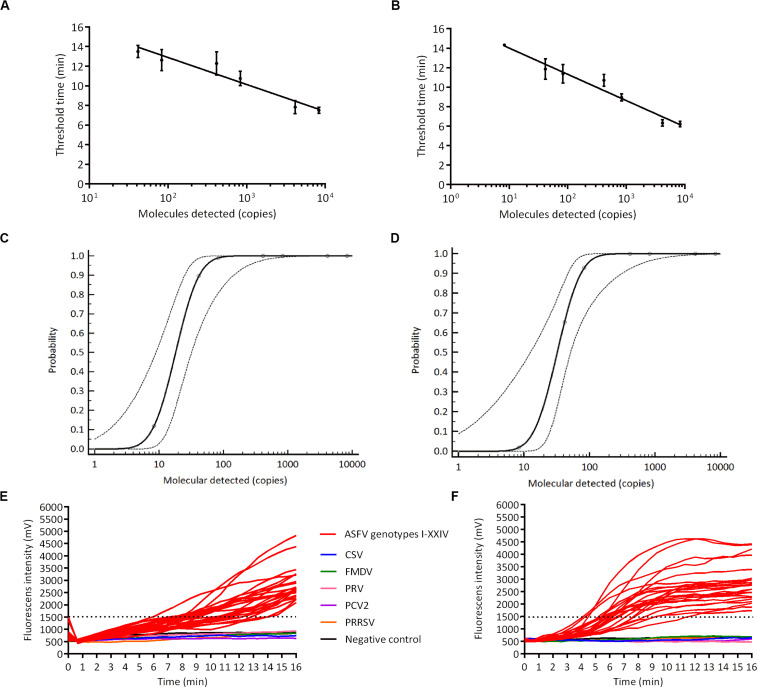
The sensitivity and specificity of recombinased-based amplification. ASFV DNA molecules after series dilution were detected by real-time RPA **(A)** and RAA **(B)** (copies per reaction). Probit regression analysis using MedCalc Software was performed on data of 8 replicates from serial dilutions by real-time RPA **(C)** and RAA **(D)**. Specificity test result of real-time RPA **(E)** and RAA **(F)** on detecting ASFV 24 genotypes, CSV, FMDV, PRV, PCV2, and PPRSV.

We analyzed the sensitivity of ASFV RPA, RAA by detecting ASFV p72 recombinant plasmids at concentrations of 8.3, 41.5, 83, 415, 830, 4150, 8300 copies/μL in eight replicates (Figures 2A,B). Our result regarding probit regression analysis showed that the detection limits of RPA and RAA at 95% probability were 93.4 copies per reaction (56.9 ∼ 619.8 copies per reaction, 95% CI) and 53.6 copies per reaction (30.7 ∼ 241.0 copies per reaction, 95% CI), respectively (Figures 2C,D). We then tested the specificity of RPA/RAA assay as well as determined the detection range of RPA/RAA method on all the genotypes of ASFV. ASFV p72 recombinant plasmids of 24 genotypes at concentration of 10^3^ ∼10^4^ copies/μL, along with CSV, FMDV, PRV, PCV2, PPRSV, were involved in the specificity test. Positive results were found among all genotypes of ASFV, whereas no cross reaction of the other microbes was shown (Figures 2E,F).

### Performance of ASFV RPA/RAA Assay on Clinical Samples and Its Comparison With Real-Time PCR Testing

**FIGURE 3:**
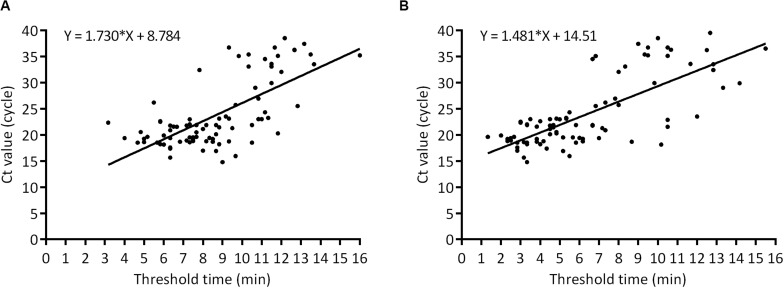
Comparison of clinical performance between the threshold time of ASFV real-time RPA (*x* axis) **(A)**, RAA (*x* axis) **(B)**, and Ct value of real-time PCR (*y* axis) on positive field samples (*n* = 88).

In order to evaluate the practical application of ASFV real-time RPA/RAA, 152 porcine samples of EDTA-blood, spleen, lung, lymph node, kidney, tonsil, liver, brain, suspected for ASFV were tested and the results were compared with that by OIE-recommended real-time PCR. Eighty eight samples were confirmed as ASFV DNA positive (Ct value, ranging from 14.77 to 39.50) while 64 as negative (Ct value, undetermined). By the detection of real-time RPA, 85 samples were identified as ASFV DNA positive [Threshold time (TT) value, ranging from 3.17 to 16.00] and 67 as negative (TT value, undetermined) (Table 1). Alternatively, the result of real-time RAA indicated 86 positive (TT value, ranging from 1.33 to 15.50) and 66 negative samples (TT value, undetermined). Linear correlation analysis revealed that, during the detection, as the Ct value of real-time PCR increased, the TT value by recombinase-based isothermal amplification present a growing trend correspondingly (Figure 3). Agreement analysis based on the detection of field samples showed that the kappa value between real-time RPA and reference real-time PCR was 0.960 (0.915 ∼ 1, 95% CI), while between real-time RAA and reference real-time PCR was 0.973 (0.936 ∼ 1, 95% CI). Additionally, in comparison to real-time PCR, the specificity and the sensitivity of RPA assay for identification of ASFV were 100% (94.40% ∼ 100%, 95% CI) and 96.59% (90.36% ∼ 99.29%, 95% CI), respectively, while those of RAA were 100% (94.40% ∼ 100%, 95% CI) and 97.73% (92.03% ∼ 99.72%, 95% CI) respectively, indicating an excellent diagnostic agreement between recombinase-based isothermal amplification and real-time PCR (Table 2).

**TABLE 1:** Detection of ASFV by RPA and RAA in clinical samples.

**Sample ID**	**Sample type**	**qPCR**		**RAA**		**RPA**	
		**Ct value**	**Result**	**TT value**	**Result**	**TT value**	**Result**
P1	Blood	19.40	+	7.00	+	4.00	+
P2	Blood	19.60	+	1.33	+	5.17	+
P3	Blood	19.90	+	2.00	+	6.00	+
P4	Blood	20.90	+	7.33	+	6.33	+
P5	Blood	21.30	+	7.17	+	9.50	+
P6	Blood	21.54	+	10.50	+	6.67	+
P7	Blood	21.80	+	4.50	+	6.33	+
P8	Blood	21.80	+	6.67	+	7.17	+
P9	Blood	21.90	+	4.67	+	10.50	+
P10	Blood	21.90	+	3.33	+	7.67	+
P11	Blood	21.90	+	6.67	+	8.67	+
P12	Blood	22.00	+	3.33	+	7.33	+
P13	Blood	22.90	+	10.50	+	7.33	+
P14	Blood	23.50	+	12.00	+	9.17	+
P15	Blood	29.00	+	13.33	+	10.67	+
P16	Blood	29.90	+	14.17	+	Undetermined	–
P17	Blood	32.05	+	8.00	+	12.00	+
P18	Blood	32.40	+	12.83	+	7.83	+
P19	Blood	33.08	+	Undetermined	-	11.50	+
P20	Blood	33.08	+	8.33	+	10.33	+
P21	Blood	33.50	+	12.83	+	13.67	+
P22	Blood	33.60	+	11.67	+	11.50	+
P23	Blood	34.50	+	6.67	+	11.17	+
P24	Blood	35.10	+	6.83	+	9.83	+
P25	Blood	35.13	+	10.50	+	11.83	+
P26	Blood	35.20	+	9.50	+	16.00	+
P27	Blood	35.38	+	9.33	+	10.33	+
P28	Blood	36.20	+	12.50	+	12.67	+
P29	Blood	36.70	+	10.50	+	9.33	+
P30	Blood	36.70	+	9.50	+	11.67	+
P31	Blood	37.43	+	9.00	+	13.17	+
P32	Blood	38.50	+	10.00	+	12.17	+
P33	Blood	39.50	+	12.67	+	Undetermined	-
P34	Brain	20.56	+	4.83	+	4.83	+
P35	Brain	22.35	+	3.00	+	3.17	+
P36	Brain	25.50	+	6.83	+	12.83	+
P37	Brain	25.70	+	8.00	+	9.67	+
P38	Brain	26.19	+	7.33	+	5.50	+
P39	Brain	26.96	+	7.83	+	10.83	+
P40	Brain	29.93	+	9.83	+	11.50	+
P41	Brain	35.39	+	Undetermined	–	13.50	+
P42	Brain	36.50	+	15.50	+	Undetermined	–
P43	Brain	36.30	+	10.67	+	12.67	+
P44	Kidney	22.60	+	4.00	+	5.83	+
P45	Kidney	23.02	+	4.50	+	10.83	+
P46	Kidney	18.20	+	3.33	+	5.83	+
P47	Kidney	19.42	+	6.00	+	8.33	+
P48	Kidney	21.11	+	4.50	+	8.00	+
P49	Kidney	21.46	+	4.67	+	8.83	+
P50	Kidney	21.88	+	5.83	+	8.17	+
P51	Kidney	22.50	+	3.00	+	5.83	+
P52	Kidney	23.00	+	3.50	+	11.00	+
P53	Kidney	23.11	+	4.83	+	8.83	+
P54	Liver	18.69	+	8.67	+	6.83	+
P55	Liver	19.40	+	2.50	+	6.33	+
P56	Liver	19.50	+	5.67	+	7.50	+
P57	Lung	17.40	+	4.33	+	6.33	+
P58	Lung	18.75	+	6.17	+	9.33	+
P59	Lung	19.26	+	6.17	+	5.00	+
P60	Lung	19.50	+	5.17	+	7.33	+
P61	Lung	20.30	+	4.83	+	11.83	+
P62	Lymph node	14.77	+	3.33	+	9.00	+
P63	Lymph node	15.96	+	5.50	+	9.67	+
P64	Lymph node	16.91	+	5.17	+	8.67	+
P65	Lymph node	18.60	+	3.17	+	5.00	+
P66	Lymph node	18.69	+	3.83	+	8.50	+
P67	Lymph node	20.13	+	4.50	+	8.67	+
P68	Lymph node	20.49	+	4.83	+	7.67	+
P69	Lymph node	21.57	+	3.83	+	6.50	+
P70	Lymph node	23.11	+	5.33	+	9.33	+
P71	Lymph node	23.24	+	5.33	+	11.33	+
P72	Lymph node	24.32	+	5.50	+	11.17	+
P73	Spleen	15.64	+	3.17	+	6.33	+
P74	Spleen	16.98	+	2.83	+	8.00	+
P75	Spleen	17.57	+	2.83	+	6.33	+
P76	Spleen	18.15	+	10.17	+	6.00	+
P77	Spleen	18.21	+	4.00	+	8.83	+
P78	Spleen	18.50	+	2.83	+	4.67	+
P79	Spleen	18.50	+	5.83	+	10.50	+
P80	Spleen	18.52	+	3.33	+	5.67	+
P81	Spleen	18.58	+	2.83	+	7.33	+
P82	Spleen	18.77	+	4.17	+	8.17	+
P83	Spleen	18.83	+	2.33	+	7.50	+
P84	Spleen	18.93	+	2.50	+	7.17	+
P85	Spleen	19.20	+	3.83	+	8.50	+
P86	Spleen	19.20	+	2.33	+	7.50	+
P87	Spleen	19.63	+	2.67	+	7.67	+
P88	Tonsil	22.99	+	5.00	+	7.33	+
N1	Blood	Undetermined	–	Undetermined	–	Undetermined	–
N2	Blood	Undetermined	–	Undetermined	–	Undetermined	–
N3	Blood	Undetermined	–	Undetermined	–	Undetermined	–
N4	Blood	Undetermined	–	Undetermined	–	Undetermined	–
N5	Blood	Undetermined	–	Undetermined	–	Undetermined	–
N6	Blood	Undetermined	–	Undetermined	–	Undetermined	–
N7	Blood	Undetermined	–	Undetermined	–	Undetermined	–
N8	Blood	Undetermined	–	Undetermined	–	Undetermined	–
N9	Blood	Undetermined	–	Undetermined	–	Undetermined	–
N10	Blood	Undetermined	–	Undetermined	–	Undetermined	–
N11	Blood	Undetermined	–	Undetermined	–	Undetermined	–
N12	Blood	Undetermined	–	Undetermined	–	Undetermined	–
N13	Blood	Undetermined	–	Undetermined	–	Undetermined	–
N14	Blood	Undetermined	–	Undetermined	–	Undetermined	–
N15	Blood	Undetermined	–	Undetermined	–	Undetermined	–
N16	Blood	Undetermined	–	Undetermined	–	Undetermined	–
N17	Blood	Undetermined	–	Undetermined	–	Undetermined	–
N18	Blood	Undetermined	–	Undetermined	–	Undetermined	–
N19	Blood	Undetermined	–	Undetermined	–	Undetermined	–
N20	Blood	Undetermined	–	Undetermined	–	Undetermined	–
N21	Blood	Undetermined	–	Undetermined	–	Undetermined	–
N22	Blood	Undetermined	–	Undetermined	–	Undetermined	–
N23	Blood	Undetermined	–	Undetermined	–	Undetermined	–
N24	Blood	Undetermined	–	Undetermined	–	Undetermined	–
N25	Blood	Undetermined	–	Undetermined	–	Undetermined	–
N26	Blood	Undetermined	–	Undetermined	–	Undetermined	–
N27	Blood	Undetermined	–	Undetermined	–	Undetermined	–
N28	Blood	Undetermined	–	Undetermined	–	Undetermined	–
N29	Blood	Undetermined	–	Undetermined	–	Undetermined	–
N30	Blood	Undetermined	–	Undetermined	–	Undetermined	–
N31	Blood	Undetermined	–	Undetermined	–	Undetermined	–
N32	Blood	Undetermined	–	Undetermined	–	Undetermined	–
N33	Blood	Undetermined	–	Undetermined	–	Undetermined	–
N34	Blood	Undetermined	–	Undetermined	–	Undetermined	–
N35	Blood	Undetermined	–	Undetermined	–	Undetermined	–
N36	Blood	Undetermined	–	Undetermined	–	Undetermined	–
N37	Blood	Undetermined	–	Undetermined	–	Undetermined	–
N38	Blood	Undetermined	–	Undetermined	–	Undetermined	–
N39	Blood	Undetermined	–	Undetermined	–	Undetermined	–
N40	Blood	Undetermined	–	Undetermined	–	Undetermined	–
N41	Blood	Undetermined	–	Undetermined	–	Undetermined	–
N42	Blood	Undetermined	–	Undetermined	–	Undetermined	–
N43	Blood	Undetermined	–	Undetermined	–	Undetermined	–
N44	Blood	Undetermined	–	Undetermined	–	Undetermined	–
N45	Blood	Undetermined	–	Undetermined	–	Undetermined	–
N46	Blood	Undetermined	–	Undetermined	–	Undetermined	–
N47	Blood	Undetermined	–	Undetermined	–	Undetermined	–
N48	Blood	Undetermined	–	Undetermined	–	Undetermined	–
N49	Blood	Undetermined	–	Undetermined	–	Undetermined	–
N50	Blood	Undetermined	–	Undetermined	–	Undetermined	–
N51	Blood	Undetermined	–	Undetermined	–	Undetermined	–
N52	Blood	Undetermined	–	Undetermined	–	Undetermined	–
N53	Blood	Undetermined	–	Undetermined	–	Undetermined	–
N54	Blood	Undetermined	–	Undetermined	–	Undetermined	–
N55	Blood	Undetermined	–	Undetermined	–	Undetermined	–
N56	Blood	Undetermined	–	Undetermined	–	Undetermined	–
N57	Kidney	Undetermined	–	Undetermined	–	Undetermined	–
N58	Kidney	Undetermined	–	Undetermined	–	Undetermined	–
N59	Kidney	Undetermined	–	Undetermined	–	Undetermined	–
N60	Lymph node	Undetermined	–	Undetermined	–	Undetermined	–
N61	Lymph node	Undetermined	–	Undetermined	–	Undetermined	–
N62	Spleen	Undetermined	–	Undetermined	–	Undetermined	–
N63	Spleen	Undetermined	–	Undetermined	–	Undetermined	–
N64	Spleen	Undetermined	–	Undetermined	–	Undetermined	–

**TABLE 2:** Diagnostic performance comparison between RPA/RAA and qPCR assays.

		**qPCR**			**Performance characteristics (%)**	
		**Positive**	**Negative**	**Total**	**Sensitivity**	**Specificity**
RPA	Positive	85	0	85	96.59% (90.36%∼99.29%, 95% CI)	100% (94.40%∼100%, 95%CI)
	Negative	3	64	67		
		88	64	152		
Agreement Kappa value: 0.960 (0.915 1, 95% CI)						
RPA	Positive	86	0	86	97.73% (92.03%∼99.72%, 95%CI)	100% (94.40%∼100%, 95% CI)
	Negative	2	64	66		
		88	64	152		

*Agreement Kappa value: 0.973 (0.936∼1, 95%CI).*

## Discussion

African swine fever represents a deadly infectious disease that causes an acute hemorrhagic fever in domestic pigs and wild boars with the mortality rate up to 100%. It currently poses major concern for global swine industry owing to the “second wave” of trans-continents transmission of ASF from Africa to Europe and Asia since 2007 in Georgia after the effective eradication except Africa and the island of Sardinia in late 1990s (Dixon et al., 2019). Unfortunately, trans-regional transportation of live pigs and pork products, the lack of good farming practice and biosecurity, movements of people and vehicles increase its further spread, which leads to costly socio-economic impact to affected countries. As there are no effective vaccines or antiviral treatment available, early detection and diagnosis at different settings, such as farms, slaughterhouses, are urgently needed as primary measures to control the disease. However, officially approved diagnostic approaches for ASF include virus isolation, fluorescent antibody test (FAT), PCR, real-time PCR, indirect fluorescent antibody (IFA), enzyme-linked immunosorbent assay (ELISA) and immunoblotting test, which generally require expensive laboratory equipment and skilled technicians (de Leon et al., 2013). This study described a rapid and reliable recombinase-based amplification assay for the detection of ASFV DNA, and evaluated its clinical potential by using two kinds of commercial kits, RPA, RAA.

Recombinase-based isothermal assay (RPA/RAA) emerges as a simple, rapid, specific and sensitive nucleic acid amplification, with the advantage of constant temperature rather than sophisticated thermocycling by PCR or real-time PCR with relatively expensive machine. It particularly assists the external application from advanced laboratories to low-resource settings (Faye et al., 2015). It has been shown that, among the development of simple and rapid detection for ASF, LAMP assay can test at least 330 genomic copies in 75 min (James et al., 2010), polymerase cross-linking spiral reaction (PCLSR) reached 720 copies/μL in 45 min (Wozniakowski et al., 2017, 2018), cross-priming amplification (CPA) identified the minimum detection limit as 200 copies within 60 min (Gao et al., 2018), chimeric DNA/LNA-based biosensor processed up to 40 samples (one sample at a time, time per analysis = 5 min) with the limits of detection being 178 copies/μL (Biagetti et al., 2018), and pen-side molecular diagnostic UPL assay based on qPCR technique still required at least 35 min (Liu et al., 2019). However, recombinase-based assay maintains detection limit of PCR but customarily shortens the reaction time from 45∼120 min of LAMP, PCLSR, CPA, PCR to 10∼30 min, correspondingly. Preliminary recombinase-based isothermal studies showed that the detection limit of ASF DNA was 108 copies per reaction (95% CI, five runs) based on RPA primers and exo probes (Wang et al., 2017a), while sensitivity was 150 copies per reaction within 15∼20 min in RPA with lateral flow detection (LFD) (Miao et al., 2019). Consistently, in our study, by testing eight replicates of serial dilutions, the analytical sensitivity of real-time RPA and RAA was further determined as 93.4 and 53.6 copies per reaction at 95% probability in 16 min, which were in a similar range (magnitude) of sensitivity and present favorable specificity as previously reported. Additionally, we evaluated the versatility on the detection of all 24 genotypes of ASFV. To the best of our knowledge, it is the first work that demonstrates a universal molecular diagnosis on all ASFV genotypes.

Recent evidence revealed that, in comparison between LAMP and CPA, although CPA reached 7.2 copies of standard ASFV plasmid, which was more sensitive than LAMP of 330 copies, yet in case of field performance, the sensitivity of CPA was 70% (14/20), lower than that of LAMP 90% (18/20), suggesting the detective potential between plasmids and clinical samples may vary (Wozniakowski et al., 2018). In this scenario, we further validate the clinical performance of recombinase-based isothermal amplification assays with 152 various kinds of field samples suspected for ASFV, including EDTA-blood, spleen, lung, lymph node, kidney, tonsil, liver, brain. Our data revealed that the specificity of both recombinased-based assays was 100%, while the sensitivity of RPA or RAA was 96.59 or 97.73%, respectively, suggesting a favorable suitability of recombinased-based assay similar to real-time PCR in clinical practice. Particularly, among 88 positive samples by OIE real-time PCR, there were 20 weak positive (Ct value ≥ 30) and 18 were tested positive by RPA/RAA. The result was also in line with and a recent review on 63 RPA-related literatures describing clinical/field trials that recombinase-based assays present a relatively high clinical specificity (100%, 51/58; 90 ∼ 99%, 5/58) and sensitivity (100%, 32/63; 80 ∼ 99%, 25/63) (Li J. et al., 2018).

In field-deployable diagnostic studies, it has been shown that Ebola virus disease (EVD) in point-of-care of Guinea primed the assembly containing a mobile glove box and a Diagnostics-in-a-Suitcase powered by a battery and solar panel and yielded a sensitivity and specificity of 100% in comparison with the real-time RT-PCR assay while saved the reaction time (Faye et al., 2015). RT-RPA mobile laboratory for Dengue virus 1–4 was established, combined with magnetic bead based total RNA extraction and a portable detection device on centrifugal lab desks that fulfilled the requirements, and all reagents involved in the mobile laboratory were cold-chain independent (Abd El Wahed et al., 2015). The utilization of a light-weight, field-deployable automatic taco^TM^ mini Nucleic Acid Automatic Extraction System, along with the insulated isothermal (ii) PCR/POCKIT^TM^ system provided an on-site diagnosis of patients with MERS-CoV infection within an hour. Notably, the overall kappa values between the two RT-iiPCR assays and the reference RT-qPCR assays were 0.96 and 0.99 (Go et al., 2017). Concomitantly, we described a simple-to-use assay format, which involves magnetic bead-based DNA extraction, pretreatment and recombinase-based isothermal amplification and processes up to 16 samples a time within 45∼50 min (Figure 1C). Our clinical result exhibited that RPA and RAA had good agreement to OIE real-time PCR (kappa value, 0.960, 0.973, respectively) and eased the manipulation of ASF diagnosis potentially for all veterinarians, veterinary officers, even inexperienced farmers in low-resource settings, without extra operation and potential carryout contamination caused by amplicon in opening tubes during result readout of agarose gel or LFD (Miao et al., 2019). Although RPA/RAA has not been so far recognized as a confirmatory diagnostic method the same as PCR or qPCR, and still requires extensive validation, it provides a potentially rapid and reliable strategy applied for early diagnosis, which is essential for subsequent early response, including disposal, movement control, disinfection, etc.

Taken together, our data on both experimental and field samples demonstrate that two recombinase-based isothermal amplification assays (RPA/RAA), coupled with field-deployable platform, contribute to a sensitive, specific and reliable tool for rapid detection of ASFV in clinic, which further facilitates screening and surveillance of ASF in the future.

## Data Availability Statement

All datasets generated for this study are included in the article/supplementary material.

## Ethics Statement

This study was performed as part of the surveillance of the ASF outbreak in China. The protocol for this study was approved by the Ethics Committee of the China Animal Health and Epidemiology Center. Clinical samples suspected for ASFV were collected by provincial Centers for Animal Disease Control and Prevention.

## Author Contributions

ZW and XW designed the experiments. XF, LL, YZ, YL, CL, QW, YD, TC, FS, CS, YW, DH, YZ, and JB performed the experiments. XF and LL analyzed the data and wrote the manuscript. All authors contributed to the article and approved the submitted version.

## Conflict of Interest

The authors declare that the research was conducted in the absence of any commercial or financial relationships that could be construed as a potential conflict of interest.

## Footnotes

1 http://www.oie.int
